# Melatonin receptor agonist protects against acute lung injury induced by ventilator through up-regulation of IL-10 production

**DOI:** 10.1186/s12931-020-1325-2

**Published:** 2020-03-06

**Authors:** Geng-Chin Wu, Chung-Kan Peng, Wen-I Liao, Hsin-Ping Pao, Kun-Lun Huang, Shi-Jye Chu

**Affiliations:** 1 The Graduate Institute of Medical Sciences, National Defense Medical Center, Taipei, Taiwan; 2 Department of Internal Medicine, Taoyuan Armed Forces General Hospital, Taoyuan, Taiwan; 3 Division of Pulmonary and Critical Care Medicine, Department of Internal Medicine, Tri-Service General Hospital, National Defense Medical Center, Taipei, Taiwan; 4 Department of Emergency Medicine, Tri-Service General Hospital, National Defense Medical Center, Taipei, Taiwan; 5 Institute of Aerospace and Undersea Medicine, National Defense Medical Center, Taipei, Taiwan; 6 Department of Internal Medicine, Tri-Service General Hospital, National Defense Medical Center, No. 325, Section 2, Chenggong Road, Neihu, Taipei, 114 Taiwan

**Keywords:** Ventilator-induced lung injury, Melatonin receptor agonist, Ramelteon, Interleukin-10

## Abstract

**Background:**

It is well known that ventilation with high volume or pressure may damage healthy lungs or worsen injured lungs. Melatonin has been reported to be effective in animal models of acute lung injury. Melatonin exerts its beneficial effects by acting as a direct antioxidant and via melatonin receptor activation. However, it is not clear whether melatonin receptor agonist has a protective effect in ventilator-induced lung injury (VILI). Therefore, in this study, we determined whether ramelteon (a melatonin receptor agonist) can attenuate VILI and explore the possible mechanism for protection.

**Methods:**

VILI was induced by high tidal volume ventilation in a rat model. The rats were randomly allotted into the following groups: control, control+melatonin, control+ramelteon, control+luzindole, VILI, VILI+luzindole, VILI + melatonin, VILI + melatonin + luzindole (melatonin receptor antagonist), VILI + ramelteon, and VILI + ramelteon + luzindole (*n* = 6 per group). The role of interleukin-10 (IL-10) in the melatonin- or ramelteon-mediated protection against VILI was also investigated.

**Results:**

Ramelteon treatment markedly reduced lung edema, serum malondialdehyde levels, the concentration of inflammatory cytokines in bronchoalveolar lavage fluid (BALF), NF-κB activation, iNOS levels, and apoptosis in the lung tissue. Additionally, ramelteon treatment significantly increased heat shock protein 70 expression in the lung tissue and IL-10 levels in BALF. The protective effect of ramelteon was mitigated by the administration of luzindole or an anti-IL-10 antibody.

**Conclusions:**

Our results suggest that a melatonin receptor agonist has a protective effect against VILI, and its protective mechanism is based on the upregulation of IL-10 production.

## Background

Acute lung injury (ALI) or acute respiratory distress syndrome (ARDS) is a relatively frequent complication in critically ill patients and is responsible for significant comorbidity and mortality levels. For five decades, multiple studies have aimed to determine the mechanisms of ARDS and reduce mortality rates. However, only lung-protective ventilation strategies with low tidal volume ventilation have been shown to reduce mortality in ARDS patients. To date, effective pharmacological therapies have not been identified for ARDS [1].

Mechanical ventilation is a life-saving tool for respiratory failure patients, but the development of ventilator-induced lung injury (VILI) is still a major problem in mechanically ventilated patients. In particular, high ventilation pressures and high tidal volumes to maintain proper oxygenation and CO_2_ elimination can cause lung damage and impair gas exchange. VILI arises from constant cyclical stretching and is characterized by alveolar wall disruption, edema formation, immune cell influx, proinflammatory cytokine release, and the excessive reactive oxygen species (ROS) production [2]. Despite advances in critical care medicine, the mortality and morbidity rates of VILI patients are still high. There is an urgent need to develop novel therapies for VILI.

Melatonin (N-acetyl-5-methoxytryptamine) is synthesized by the pineal gland in mammals and also by other nonendocrine organs, such as the skin, gut, and immune system. It has multiple properties, including antiapoptotic, antioxidative, and anti-inflammatory effects. Melatonin exerts its physiological effects via receptor-dependent and receptor-independent signaling cascades [3, 4]. The role of melatonin receptors in organ protection has been investigated. The activation of melatonin receptors has been reported to protect against myocardial ischemia/reperfusion injury and cyclosporine A cardiotoxicity [5, 6]. In addition, melatonin also exerts antiapoptotic effects via melatonin receptor interactions [4]. Furthermore, melatonin receptors mediate improvements in survival after polymicrobial sepsis in rats and mice [7].

IL-10 is an anti-inflammatory cytokine with strong effects on numerous cell types, particularly circulating and resident immune cells and epithelial cells. Previous studies have shown that lung cells subjected to stretching have reduced IL-10 production [8]. In a model of VILI, IL-10 inhalation reduced the concentrations of macrophage inflammatory protein-2 and IL-1β in bronchoalveolar lavage fluid (BALF) and plasma [2]. In addition, IL-10 is produced by Th2 cells.

There is increasing evidence that melatonin modulates immune responses and exhibits marked modulatory functions in effector T cells [9]. Investigations have revealed that melatonin enhances the secretion of IL-4 and IL-10, acting in the Th2-like immune response [9]. In addition, the regulation of T cell functions by melatonin occurs via melatonin receptors because the effect of melatonin on the activation of T cell functions is suppressed by luzindole (a melatonin receptor antagonist) treatment [9].

Ramelteon is a potent and highly selective agonist of the high-affinity melatonin receptor 1 (MT1) and MT2 receptors and has antioxidative and anti-inflammatory effects [10–12]. However, the role of melatonin receptor agonist in experimental VILI has not yet been investigated. Thus, the purpose of this study was to determine whether ramelteon provided protection in a rat model of VILI and its protective mechanism was via regulating IL-10 production.

## Methods

### Animals

Experiments were performed on 6- to 8-week-old male Sprague Dawley rats weighing 350 ± 20 g. The animal care and experimental protocol were approved by the Animal Review Committee at the National Defense Medical Center in Taipei, Taiwan.

### Experimental protocols

The rats were anesthetized with an intraperitoneal pentobarbital (80 mg/kg) injection. Catheters were inserted into the femoral artery and vein, and a tracheostomy was performed. Anesthesia was maintained with intravenous pentobarbital at 18 mg/hr. The femoral artery catheter was inserted to collect biological signals to monitor the mean arterial pressure and heart rate continuously. The rats were placed in a supine position and connected to a ventilator (Model 7025, Ugo Basile, Varese, Italy). Then, the rats were ventilated with volume control for 5 h with an inspired oxygen fraction of 0.21, positive end-expiratory pressure of 2 cmH_2_O, and respiratory rate of 70 breaths/min. The rats were randomly assigned to receive mechanical ventilation with either a tidal volume of 20 mL/kg (high tidal volume) or 10 mL/kg (low tidal volume). A positive end-expiratory pressure of 2 cmH_2_O was used in both cases [13].

The rats were randomly allocated into 10 groups (*n* = 6 per group): group 1 (control with low tidal volume ventilation, 10 mL/kg); group 2 (control+ 20 mg/kg melatonin); group 3 (control+ 5 mg/kg ramelteon); group 4 (control+ 2 mg/kg luzindole); group 5 (VILI with high tidal volume ventilation, 20 mL/kg); group 6 (VILI + 2 mg/kg luzindole); group 7 (VILI + 20 mg/kg melatonin); group 8 (VILI + 20 mg/kg melatonin + 2 mg/kg luzindole); group 9 (VILI + 5 mg/kg ramelteon); and group 10 (VILI + 5 mg/kg ramelteon + 2 mg/kg luzindole). We performed another animal study to evaluate the role of IL-10 in melatonin- and ramelteon-mediated protection against VILI. The rats were randomly allocated into 8 groups: group 1 (control); group 2 (control + 10 μg anti-IL-10 antibody); group 3 (VILI); group 4 (VILI + 10 μg anti-IL-10 antibody); group 5 (VILI + 20 mg/kg melatonin); group 6 (VILI + 10 μg anti-IL-10 antibody + 20 mg/kg melatonin); group 7 (VILI + 5 mg/kg ramelteon); and group 8 (VILI + 10 μg anti-IL-10 antibody + 5 mg/kg ramelteon). Melatonin, ramelteon, luzindole, or anti-IL-10 antibody treatment was administered via femoral vein injection 30 min before VILI. Ramelteon (LKT laboratories Inc., USA) and melatonin (Sigma-Aldrich, USA) were dissolved in absolute ethanol. The resulting solution was diluted with saline until a concentration of 0.3% ethanol in saline was obtained. Luzindole (Tocris, England) was dissolved in dmethyl sulfoxide (DMSO). The resulting solution was diluted with saline until a concentration of 0.15% DMSO in saline was obtained. The concentrations of ethanol and DMSO in control group did not cause lung injury (supplement Figure 1). The anti-IL-10 antibody (Abcam, Cambridge, MA, USA) was dissolved in 0.9% saline. The doses of various agents in this study were chosen according to previous studies [14–17].

### Arterial blood gas analysis

Blood (0.5 mL) was drawn from the right femoral artery at the beginning and at hourly intervals in each experiment. Arterial blood gas levels were immediately measured with a blood gas analyzer (ABL 800 FLEX blood gas analyzer, Radiometer, Denmark).

### Measurement of the lung weight/body weight (LW/BW) and wet/dry (W/D) weight ratios

At the end of the experiments, the right lung was removed from the hilar region, and the wet weight was obtained for the calculation of the LW/BW ratio. The middle lobe of the right lung was excised, and the wet weight was recorded. The lobe was then placed in an incubator at 60 °C for 48 h to obtain the dry weight. The W/D weight ratios were calculated by dividing the wet weight by the dry weight.

### Histopathological analysis

The lung tissues were fixed, sectioned, stained with hematoxylin and eosin, and viewed under a light microscope. The scoring was performed by two pathologists who were blinded to the experimental conditions. A minimum of 10 random areas were examined at a magnification of × 400. Each histological characteristic was graded according to the following four-point scale: 0, no damage; 1, mild damage; 2, moderate damage; 3, severe damage according to the following morphological characteristics: (1) infiltration or aggregation of neutrophils, and (2) the thickness of the alveolar walls [18, 19]. The resulting scores were summed to represent the lung injury score. The numbers of polymorphonuclear neutrophils and lung injury score in the lung tissue were also analyzed.

### Determination of the serum malondialdehyde (MDA) level

The extent of serum lipid peroxidation was determined by measuring the concentration of thiobarbituric acid-reactive substances (TBARS) using a TBARS ELISA kit (Cayman Chemical Co., Ann Arbor, MI, USA). Standards or samples were boiled for 1 hour at 100 °C and centrifuged at 16,000×*g* for 10 min. The absorbance was recorded at 532 nm.

### Protein carbonyl content in lung tissue

The oxidative damage to proteins was assessed by determining carbonyl groups based on a reaction with dinitrophenylhydrazine, as previously described [18]. The carbonyl content was determined from the absorbance at 370 nm while assuming a molar absorption coefficient of 220,000 M^− 1^. The result was expressed as the concentration of carbonyl derivatives in the protein (nmol carbonyl/mg protein).

### Immunohistochemical analyses

Immunohistochemical staining was performed to identify myeloperoxidase (MPO) in the lung tissue. Paraffin-embedded lung tissue sections fixed with formalin were deparaffinized and pretreated for antigen retrieval. Endogenous peroxidase was blocked by incubating in 3% H_2_O_2_ in methanol for 15 min. Immunostaining of the lung sections was performed using an anti-MPO-specific antibody (1:1000; Cell Signaling Technology, Danvers, MA, USA). After being washed, the slides were sequentially incubated with rat tissue-specific a horseradish peroxidase-conjugated anti-rabbit antibody (Nichirei Corporation Tokyo, Japan) for 30 min, and the lung tissue sections were counterstained with hematoxylin [18, 19].

### Measurement of protein and cytokine levels in BALF

The levels of protein,tumor necrosis factor-α (TNF-α), IL-1β, IL-6, CXCL-1, and IL-10 in BALF were measured after the experiment. The protein concentration in the supernatant was determined using a bicinchoninic acid protein assay kit (Pierce, Rockford, IL, USA). The levels of TNF-α, IL-1β, IL-6, CXCL-1, and IL-10 were determined using an ELISA kit (R&D Systems Inc., Minneapolis, MN, USA) [18, 19].

### Immunoblotting

Immunoblotting was performed as described previously [18]. Immunoblotting was carried out with antibodies against STAT3, B-cell lymphoma (Bcl)-2, poly (ADP-ribose) polymerase (PARP), caspase-3, NF-κB p65, inhibitor of NF-κB (IκB)-α (Cell Signaling Technology, USA), iNOS (BD Biosciences, USA), heat shock protein 70 (Hsp70; Santa Cruz Biotechnology, USA), and β-actin (for cytoplasmic proteins, diluted 1:10,000; Sigma-Aldrich, USA).

### Statistical analysis

The data are expressed as the mean ± SD. Significant differences between groups were determined with a one-way ANOVA. Scheffe’s test was used for post hoc comparisons. Significance was considered to be present at *p*-values < 0.05. All of the graphs and statistics were generated with GraphPad Prism 5 (GraphPad Software, San Diego, CA, USA).

## Results

### Effect of melatonin and ramelteon on oxygenation

The PaO_2_ concentration at 5 h after VILI was significantly higher in the VILI + melatonin and VILI + ramelteon groups than the VILI group. However, the increased oxygenation concentration in the VILI + melatonin and VILI + ramelteon groups was reduced by the administration of luzindole (Table 1).

**Table 1:** Effect of melatonin and ramelteon on oxygenation

Treatment group	PaO_2_
Control	120.3 ± 8.8
Control + melatonin	117.5 ± 10.3
Control + Ramelteon	117.1 ± 22.5
Control + luzindole	115.8 ± 14.5
VILI	65.6 ± 20.6^*^
VILI+ luzindole	64.7 ± 24.3^*^
VILI+ Melatonin	116.7 ± 21.0^**#**^
VILI + Melatonin + luzindole	80.4 ± 13.7^*^
VILI + Ramelteon	105.7 ± 22.3^**#**^
VILI + Ramelteon + luzindole	79.8 ± 13.9^*^

The data are expressed as the mean ± SD. *Significantly different from the control (*P* < 0.05); ^*#*^ Significantly different from the VILI group (*P* < 0.05)

### Effect of melatonin and ramelteon on lung edema

**Fig. 1:**
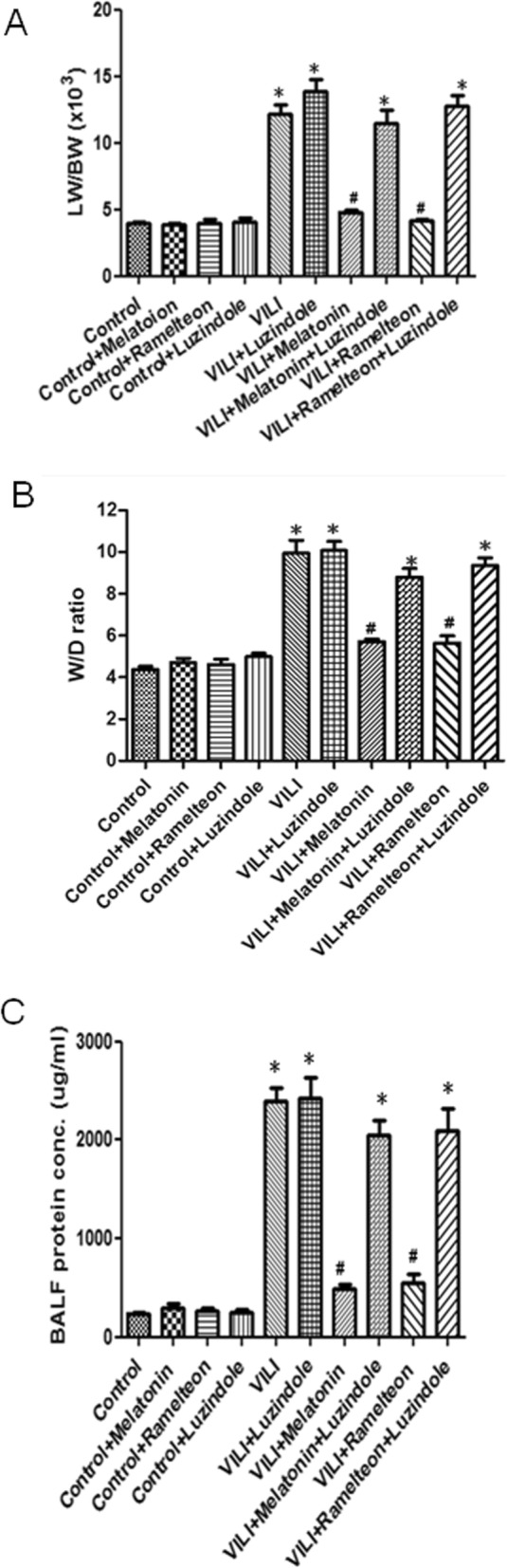
Effect of melatonin and ramelteon on lung edema. VILI significantly increased the lung weight/body weight ratio (**a**), W/D weight ratio (**b**), and protein levels in BALF (**c**). Melatonin or ramelteon treatment significantly reduced these increases. The protective effect of melatonin and ramelteon was abrogated by luzindole treatment. The data are expressed as the mean ± SD (*n* = 6 per group). *Significantly different from the control (*p*-values < 0.05); ^*#*^significantly different from the VILI group (*p* < 0.05)

As shown in Fig. 1a-c, the LW/BW and W/D weight ratios and the protein levels in BALF were significantly increased in the VILI group compared to the control group. Moreover, melatonin or ramelteon treatment significantly reduced these increases. However, these effects were abolished by the administration of luzindole.

### Effect of melatonin and ramelteon on serum MDA levels, carbonyl content, and MPO-positive cell staining in lung tissue

**Fig. 2:**
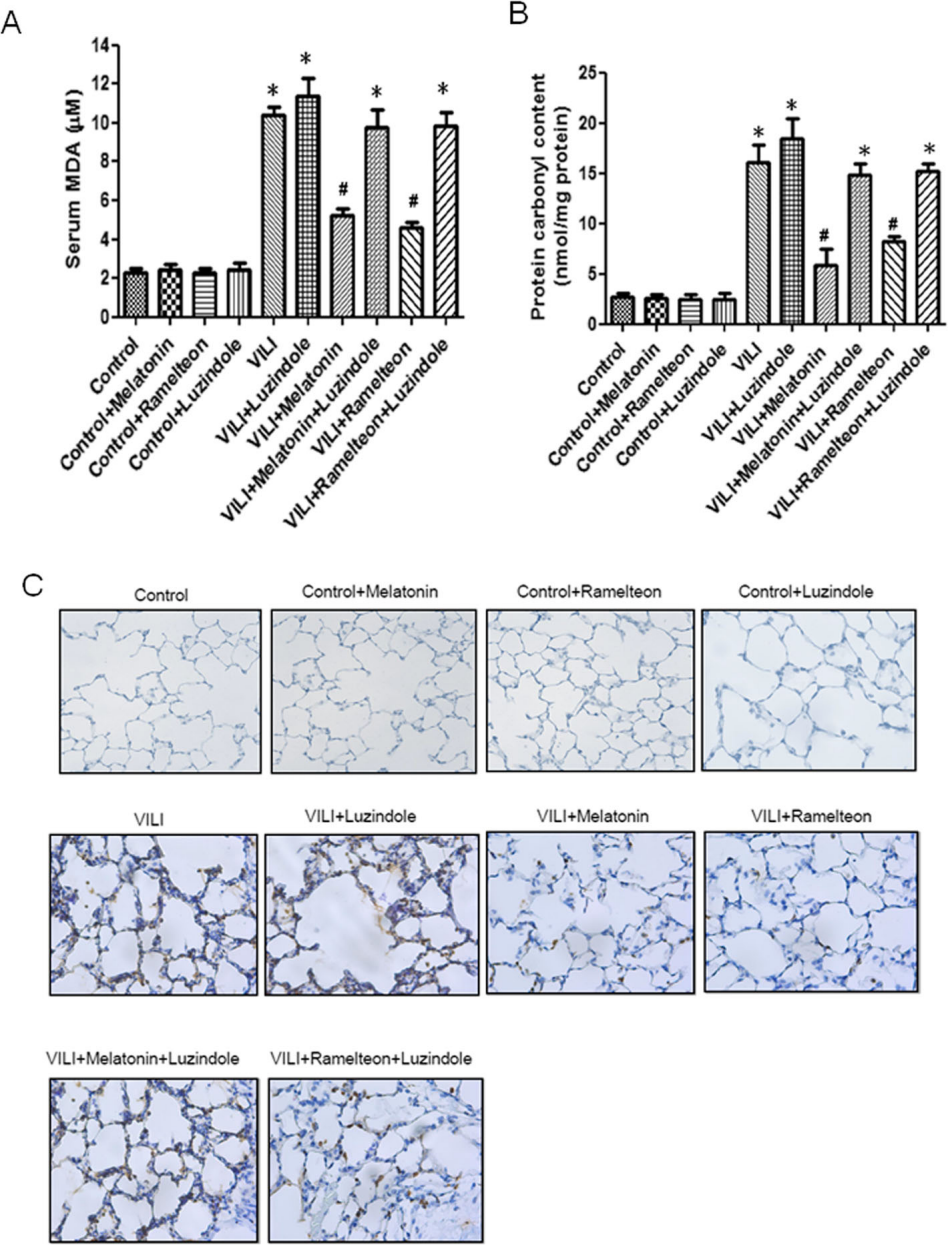
Effect of melatonin and ramelteon on serum malondialdehyde (MDA) levels, carbonyl content and myeloperoxidase (MPO)-positive cell numbers in lung tissue. Serum MDA levels (**a**), carbonyl content (**b**) and MPO-positive cell numbers in lung tissue (**c**) were significantly increased in the VILI group. Melatonin or ramelteon treatment significantly attenuated these increases. The protective effect of melatonin and ramelteon was abrogated by luzindole treatment. The data are expressed as the mean ± SD (*n* = 6 per group). *Significantly different from the control (*p*-values < 0.05); ^*#*^significantly different from the VILI group (*p* < 0.05)

In comparison to the control group, the VILI group had significantly increased serum MDA levels, carbonyl content in the lung tissue, and numbers of MPO-positive cells in the lung tissue. Treatment with melatonin or ramelteon significantly diminished these increases. In contrast, the reduction in serum MDA levels, carbonyl content, and numbers of MPO-positive cells in the lung tissue by melatonin or ramelteon was abolished by treatment with luzindole (Fig. 2a-c).

### Effect of melatonin and ramelteon on TNF-α, IL-1β, IL-6, and CXCL-1 concentrations in BALF

**Fig. 3:**
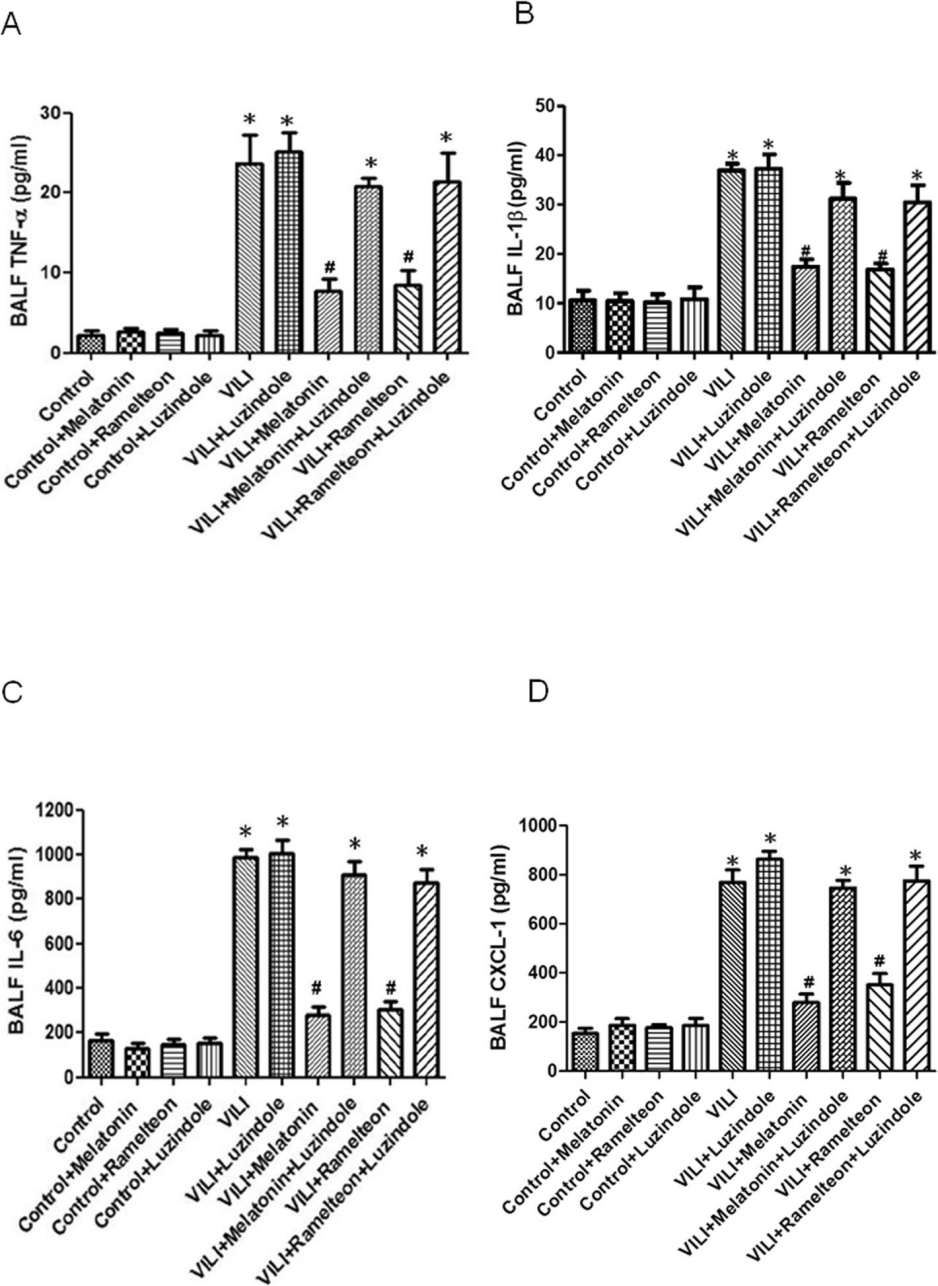
Effect of melatonin and ramelteon on TNF-α, IL-1β, IL-6, and CXCL-1 concentrations in BALF. TNF-α (**a**), IL-1β (**b**), IL-6 (C), and CXCL-1 (**d**) levels in BALF increased significantly in the VILI group. The increases in the BALF protein concentrations were significantly attenuated by treatment with melatonin or ramelteon. The protective effect of melatonin and ramelteon was abrogated by luzindole treatment. The data are expressed as the mean ± SD (*n* = 6 per group). *Significantly different from the control (*p* < 0.05); ^*#*^significantly different from the VILI group (*p* < 0.05)

As shown in Fig. 3a-d, the BALF levels of TNF-α, IL-1β, IL-6, and CXCL-1 were significantly increased in the VILI group compared with the control group. Melatonin or ramelteon treatment significantly inhibited the VILI-induced increases in TNF-α, IL-1β, IL-6, and CXCL-1 levels in BALF. However, the protective effect of melatonin or ramelteon was abolished by treatment with luzindole.

### Effect of melatonin and ramelteon on IL-10 and STAT3 expression

**Fig. 4:**
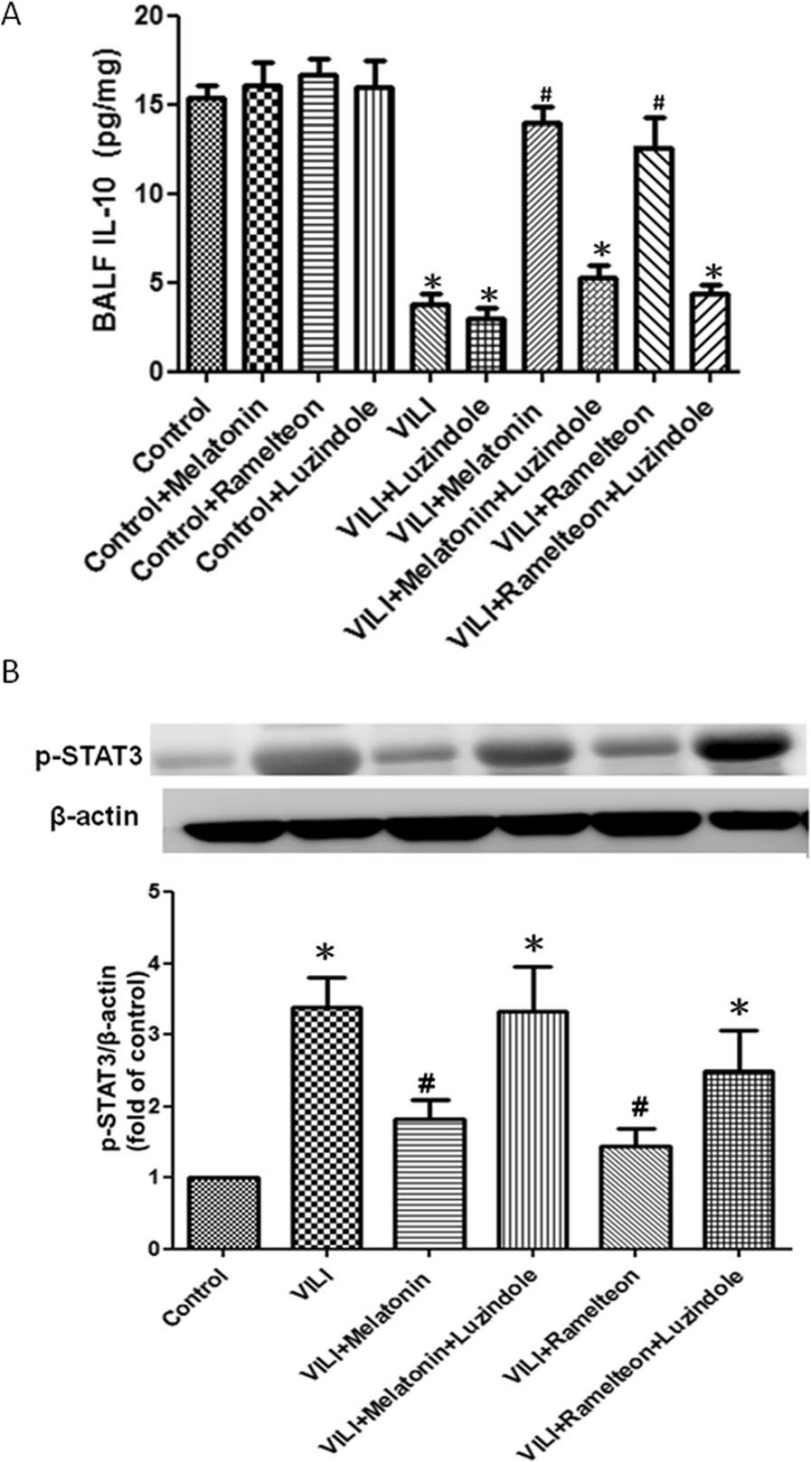
Effect of melatonin and ramelteon on IL-10 levels in BALF and STAT3 expression in the lung tissue. The p-STAT 3 level in lung tissue was determined by western blotting. β-Actin served as a loading control for cytoplasmic proteins. VILI significantly decreased IL-10 levels in BALF (**a**) and increased p-STAT3 protein expression in the lung tissue (**b**). Melatonin or ramelteon treatment significantly reversed this phenomenon. When luzindole was added, the protective effect of melatonin and ramelteon was blocked. Data are expressed as the mean ± SD (*n* = 6 per group). *Significantly different from the control (*p* < 0.05); ^*#*^significantly different from the VILI group (p < 0.05)

Compared with the control group, the VILI group had significantly lower IL-10 levels in BALF and greater STAT3 phosphorylation levels in the lung tissue. However, the decreased IL-10 level in the VILI group was significantly elevated by melatonin or ramelteon treatment. The increased STAT3 phosphorylation in the VILI group was significantly mitigated by melatonin or ramelteon treatment. Furthermore, the administration of luzindole blocked the effects of melatonin and ramelteon (Fig. 4a-b).

### Effect of melatonin and ramelteon on iNOS expression

**Fig. 5:**
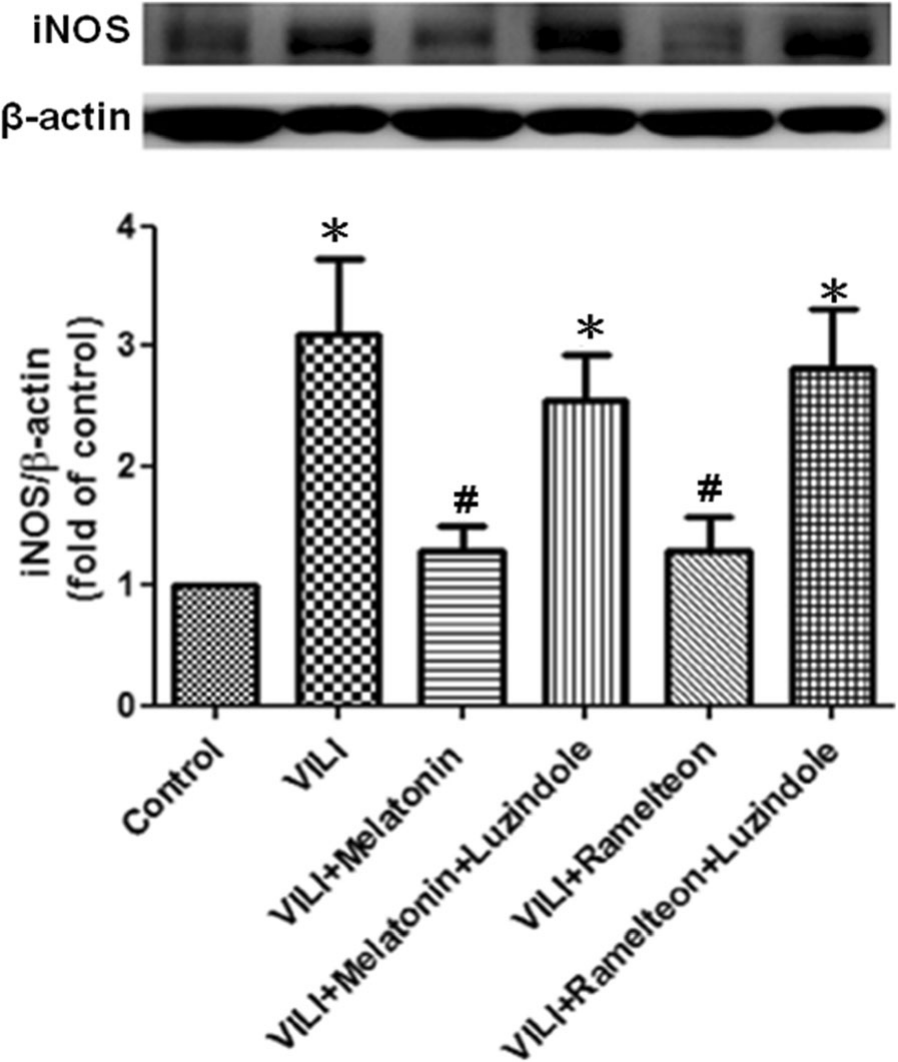
Effect of melatonin and ramelteon on iNOS expression. iNOS levels in the lung tissue were determined by western blotting. β-Actin served as a loading control for cytoplasmic proteins. VILI significantly increased iNOS protein expression. Melatonin or ramelteon treatment significantly attenuated the increase. When luzindole was added, the protective effect of melatonin and ramelteon was blocked. The data are expressed as the mean ± SD (*n* = 6 per group). *Significantly different from the control (p-values < 0.05); ^*#*^ significantly different from the VILI group (*p* < 0.05)

The iNOS level of the lung tissue was significantly increased after VILI induction, but the increase was significantly suppressed by melatonin or ramelteon treatment. In contrast, treatment with luzindole counteracted the protective effect of melatonin and ramelteon (Fig. 5).

### Effect of melatonin and ramelteon on lung pathology

**Fig. 6:**
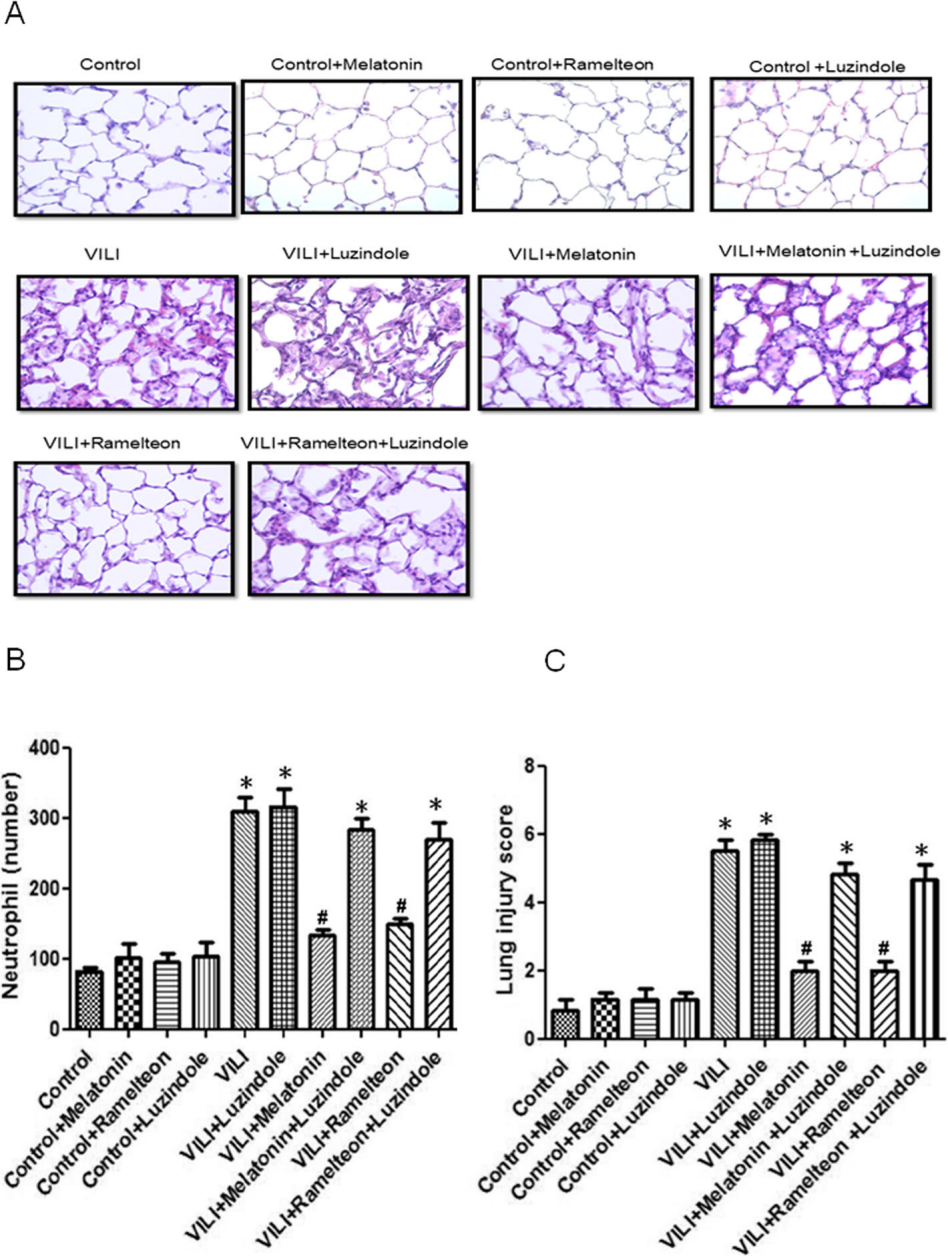
Effect of melatonin and ramelteon on lung pathology. **a** Hematoxylin and eosin staining analysis of lung pathological injury. Representative photomicrographs were taken at a magnification of × 400. **b** The numbers of neutrophils per high-power field (× 400 magnification), and **c** lung injury score. Histological evaluation of lung tissues showed that neutrophil infiltration and the lung injury score were increased in the VILI group. Melatonin or ramelteon treatment significantly attenuated these histopathological changes, but the protective effect of melatonin and ramelteon was abrogated by luzindole treatment. The data are expressed as the mean ± SD (*n* = 6 per group). *Significantly different from the control (*p* < 0.05); *#*significantly different from the VILI group (p < 0.05)

As shown in Fig. 6a, the control group exhibited normal lung tissue structures. In contrast, severe lung damage was observed in the VILI group, as indicated by extensive interstitial edema and leucocyte infiltration. Melatonin or ramelteon treatment significantly reduced the histological changes (Fig. 6a), neutrophil infiltration (Fig. 6b), and lung injury scores (Fig. 6c) in the VILI group. However, the protective effects were abolished by luzindole treatment.

### Effect of melatonin and ramelteon on the NF-κB signaling pathway

**Fig. 7:**
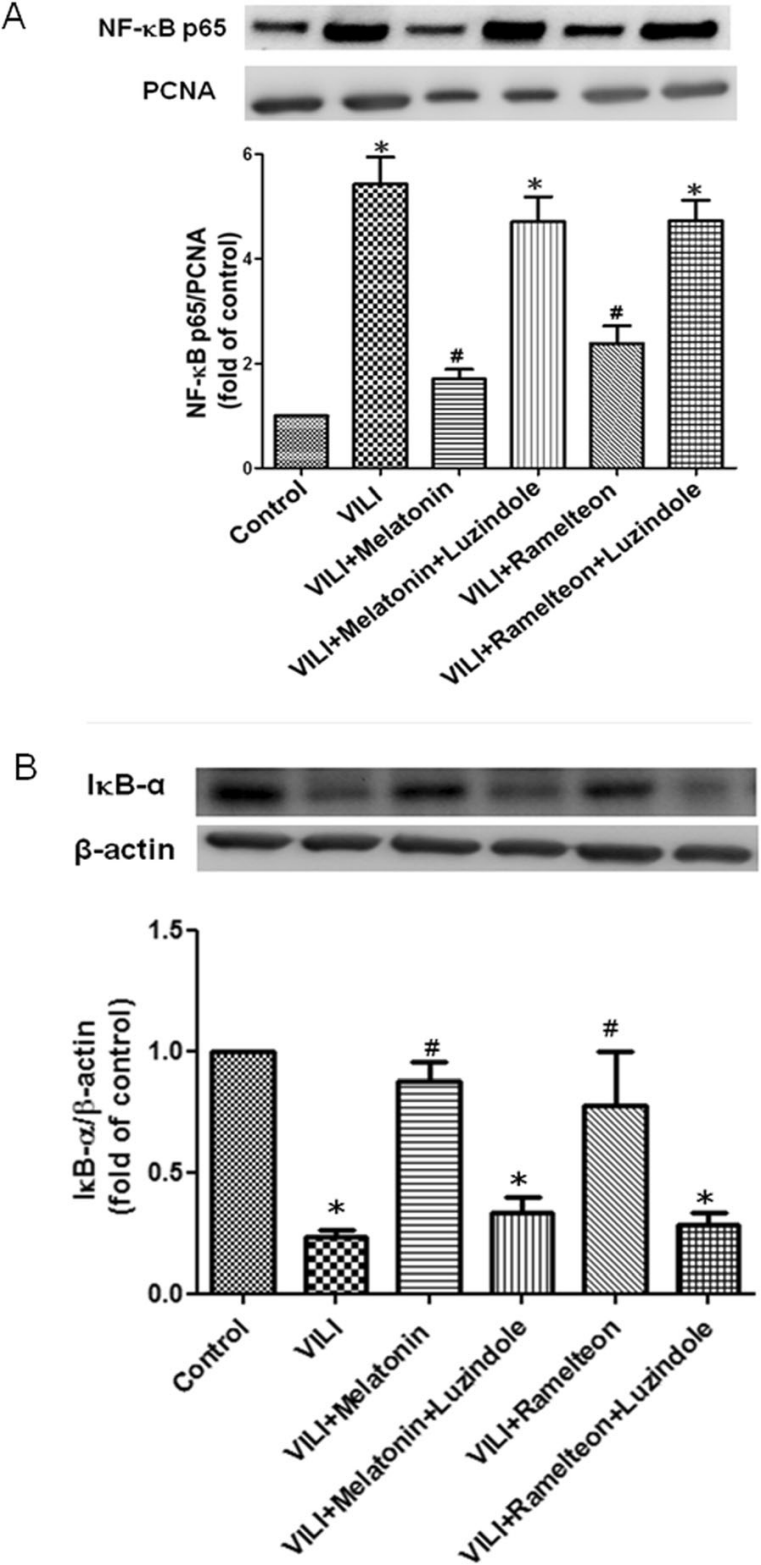
Effect of melatonin and ramelteon on the NF-κB signaling pathway. **a** The NF-κB p65 level and **b** IκBα level in the lung tissue were determined by western blotting. PCNA and β-actin served as loading controls for nuclear and cytoplasmic proteins, respectively. Representative blots are shown. Melatonin or ramelteon treatment reduced NF-κB p65 levels and increased IκB-α levels in VILI. When luzindole was added, the protective effect was blocked. The data are expressed as the mean ± SD (*n* = 6 per group). *Significantly different from the control (*p* < 0.05); ^*#*^significantly different from the VILI group (p < 0.05)

The protein level of NF-κB p65 in the nucleolus was significantly increased, but the protein level of IκB-α in the cytoplasm was significantly decreased in the VILI group compared with the control group (Fig. 7a-b). Melatonin or ramelteon treatment restored the suppressed IκB-α expression and reduced nuclear NF-κB p65 expression. Treatment with luzindole counteracted the protective effect of melatonin and ramelteon (Fig. 7a-b).

### Effect of melatonin and ramelteon on apoptosis

**Fig. 8:**
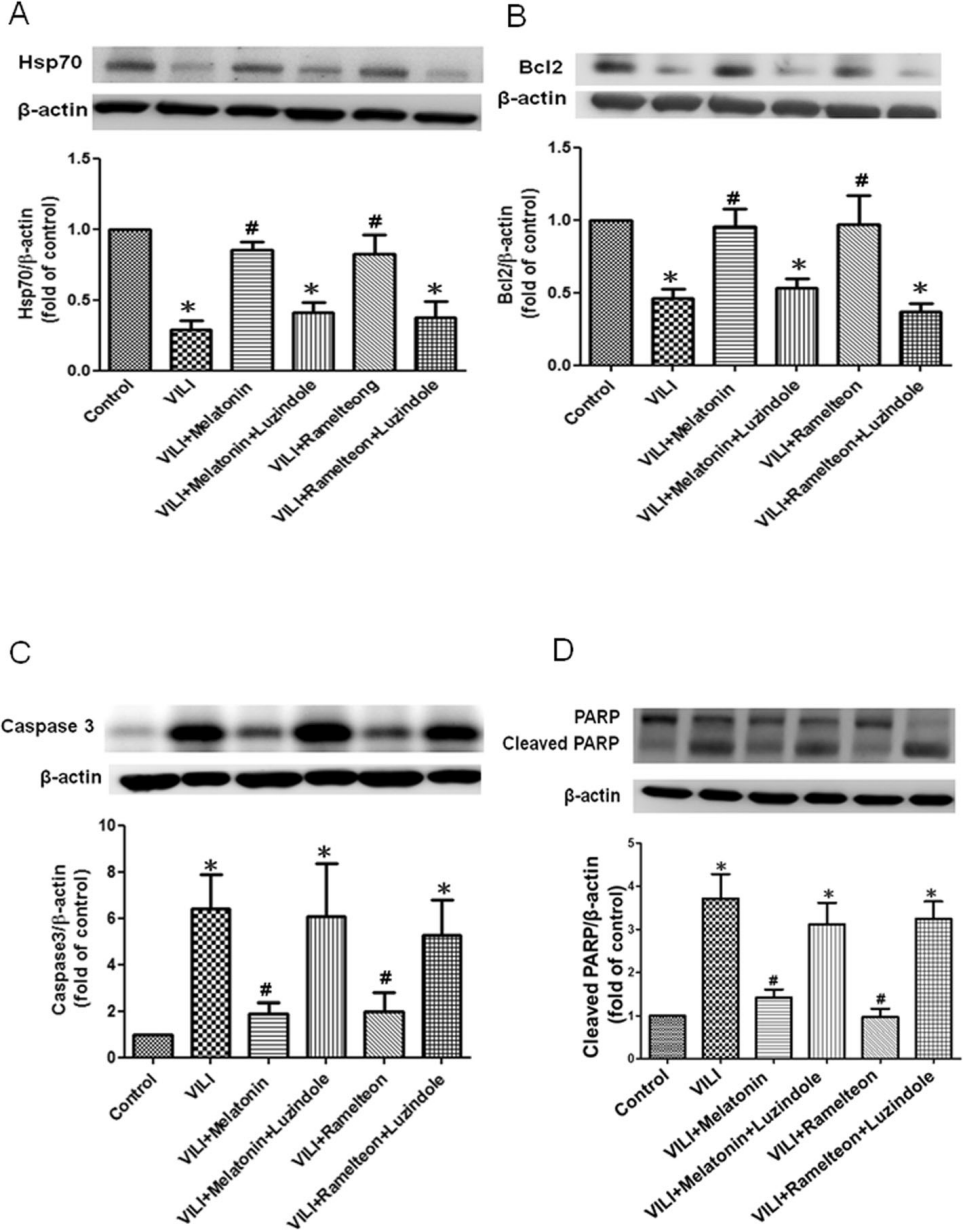
Effect of melatonin and ramelteon on apoptosis. Hsp70 (**a**), Bcl-2 (**b**), caspase-3 (**c**), and cleaved PARP (**d**) levels in the lung tissue were determined by western blotting. Melatonin or ramelteon treatment increased Hsp70 and Bcl-2 levels and reduced caspase-3 and cleaved PARP levels in VILI. When luzindole was added, the protective effect was blocked. The data are expressed as the mean ± SD (*n* = 6 per group). *Significantly different from the control (*p* < 0.05); ^*#*^significantly different from the VILI group (p < 0.05)

The Hsp70 (Fig. 8a) and Bcl-2 (Fig. 8b) protein levels in the lung tissue were substantially lower in the VILI group than the control group. However, they were significantly increased upon melatonin or ramelteon treatment. The protective effect was abolished by treatment with luzindole. The expression of caspase-3 and cleaved PARP in the lung tissue was higher in the VILI group than the control group, but melatonin or ramelteon treatment attenuated the increased expression of caspase-3 and cleaved PARP in the VILI group. Similarly, the protective effects of melatonin and ramelteon were abolished by treatment with luzindole (Fig. 8c-d).

### Effect of an anti-IL-10 antibody on the melatonin- and ramelteon-mediated the levels of BALF IL-10

**Fig. 9:**
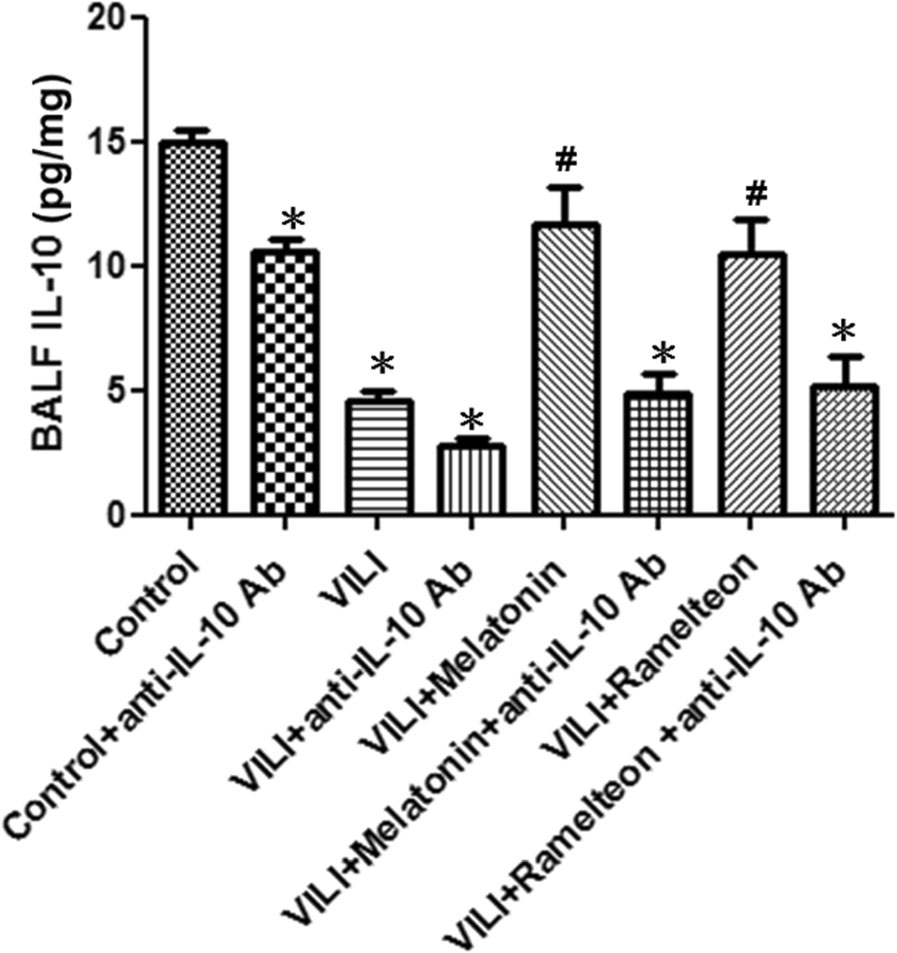
Effect of an anti-IL-10 antibody on melatonin- and ramelteon-mediated BALF IL-10 levels. VILI significantly decreased IL-10 levels in BALF. Melatonin or ramelteon treatment significantly reversed this phenomenon. When an anti-IL-10 antibody was administered, the protective effect of melatonin and ramelteon was blocked. Data are expressed as the mean ± SD (n = 6 per group). *Significantly different from the control (p < 0.05); ^*#*^significantly different from the VILI group (p < 0.05)

Compared with the control group, the control + anti-IL-10 antibody group had significantly lower IL-10 level in BALF. Moreover, VILI group had significantly lower IL-10 level in BALF. The decreased BALF IL-10 level in the VILI group was significantly elevated by melatonin or ramelteon treatment. However, the administration of anti-IL-10 antibody blocked the effects of melatonin and ramelteon (Fig. 9).

### Effect of an anti-IL-10 antibody on the melatonin- and ramelteon-mediated protection against lung edema

**Fig. 10:**
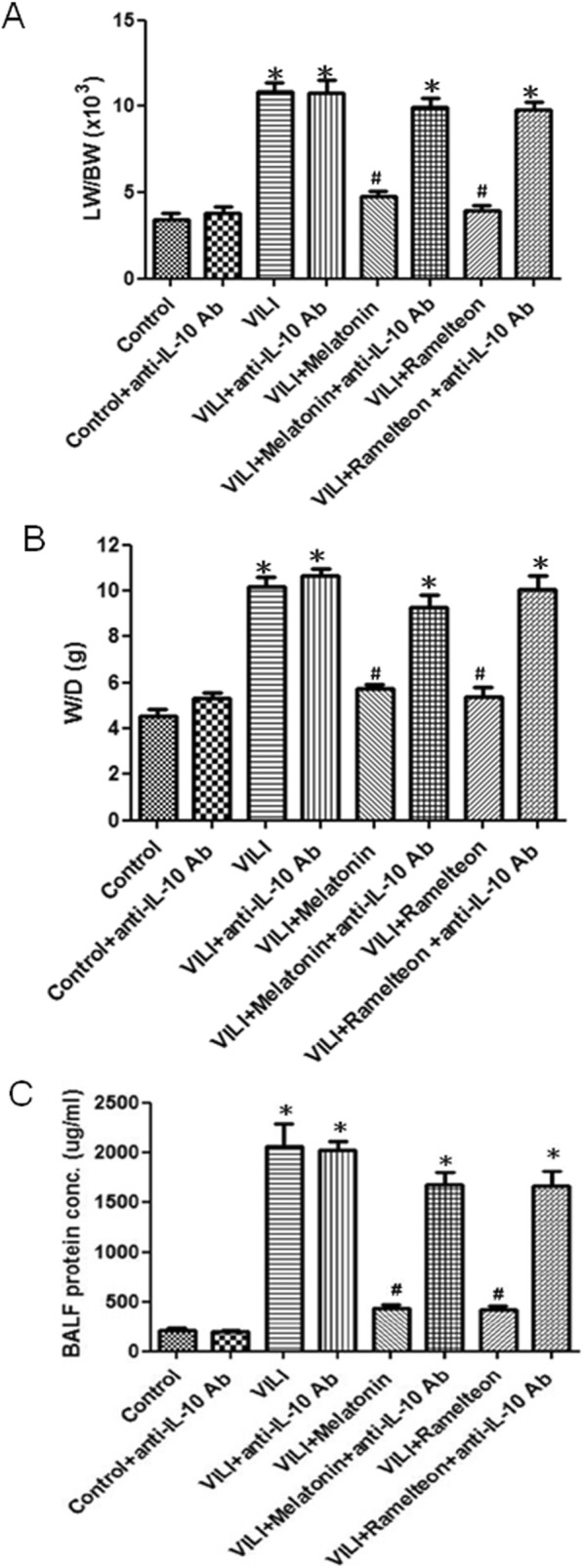
Effect of anti-IL-10 antibody on melatonin- and ramelteon-mediated VILI protection against lung edema. **a** Lung weight/body weight ratio. **b** W/D weight ratio. **c** Protein levels in BALF. Melatonin or ramelteon treatment significantly attenuated the increase in LW/BW and W/D weight ratios and protein levels in VILI. When an anti-IL-10 antibody was administered, the protective effect was blocked. The data are expressed as the mean ± SD (n = 6 per group). *Significantly different from the control (p < 0.05); ^*#*^significantly different from the VILI group (p < 0.05)

Compared with the control group, the VILI group had significantly increased LW/BW and W/D weight ratios and protein levels in BALF. Melatonin or ramelteon treatment significantly reduced these increases. However, anti-IL-10 antibody administration blocked the protective effect of melatonin and ramelteon against VILI induced lung edema (Fig. 10a-c).

### Effect of an anti-IL-10 antibody on the melatonin- and ramelteon-mediated cytokines concentrations in BALF

**Fig. 11:**
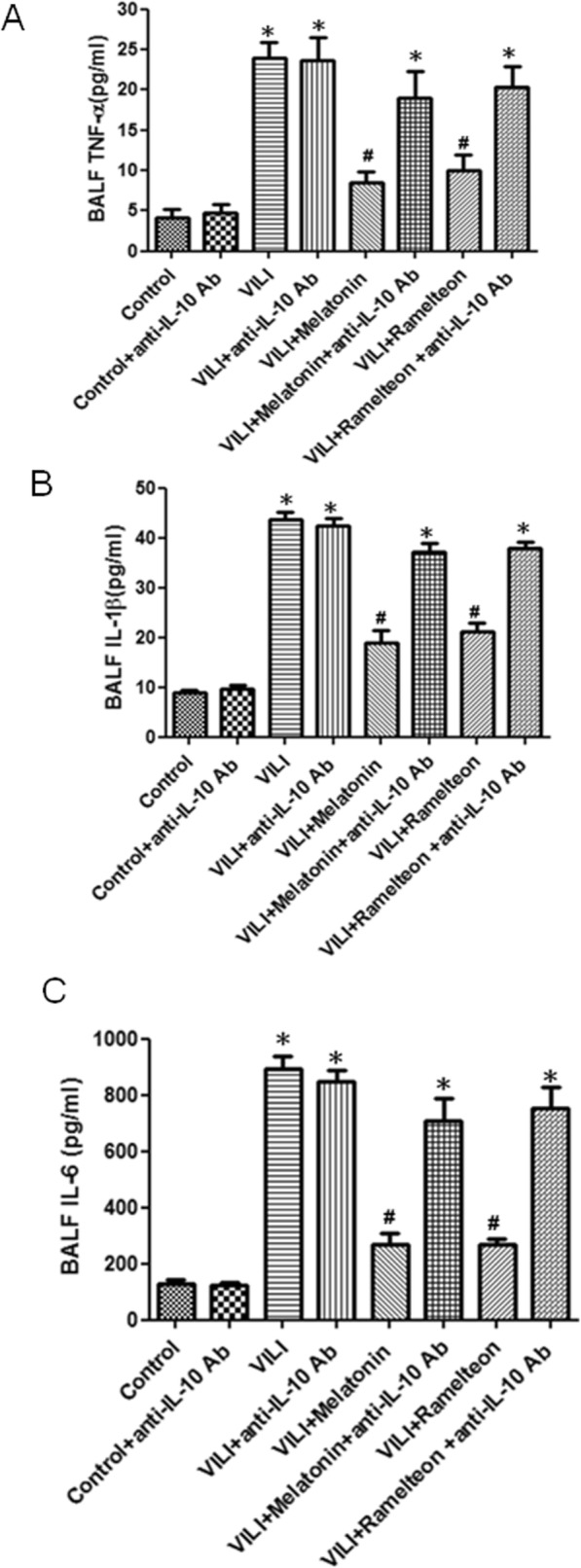
Effect of an anti-IL-10 antibody on the melatonin- and ramelteon-mediated cytokines concentrations in BALF. TNF-α (**a**), IL-1β (**b**), and IL-6 (**c**) levels in BALF increased significantly in the VILI group. The increases in the BALF protein concentrations were significantly attenuated by treatment with melatonin or ramelteon. The protective effect of melatonin and ramelteon was abrogated by anti-IL-10 antibody treatment. The data are expressed as the mean ± SD (n = 6 per group). *Significantly different from the control (p < 0.05); ^*#*^significantly different from the VILI group (p < 0.05)

As shown in (Fig. 11a-c), melatonin or ramelteon treatment significantly inhibited the VILI-induced increases in TNF-α, IL-1β, and IL-6 levels in BALF. However, the protective effect of melatonin or ramelteon was abolished by treatment with anti-IL-10 antibody.

### Effect of an anti-IL-10 antibody on the melatonin- and ramelteon-mediated lung pathology

**Fig. 12:**
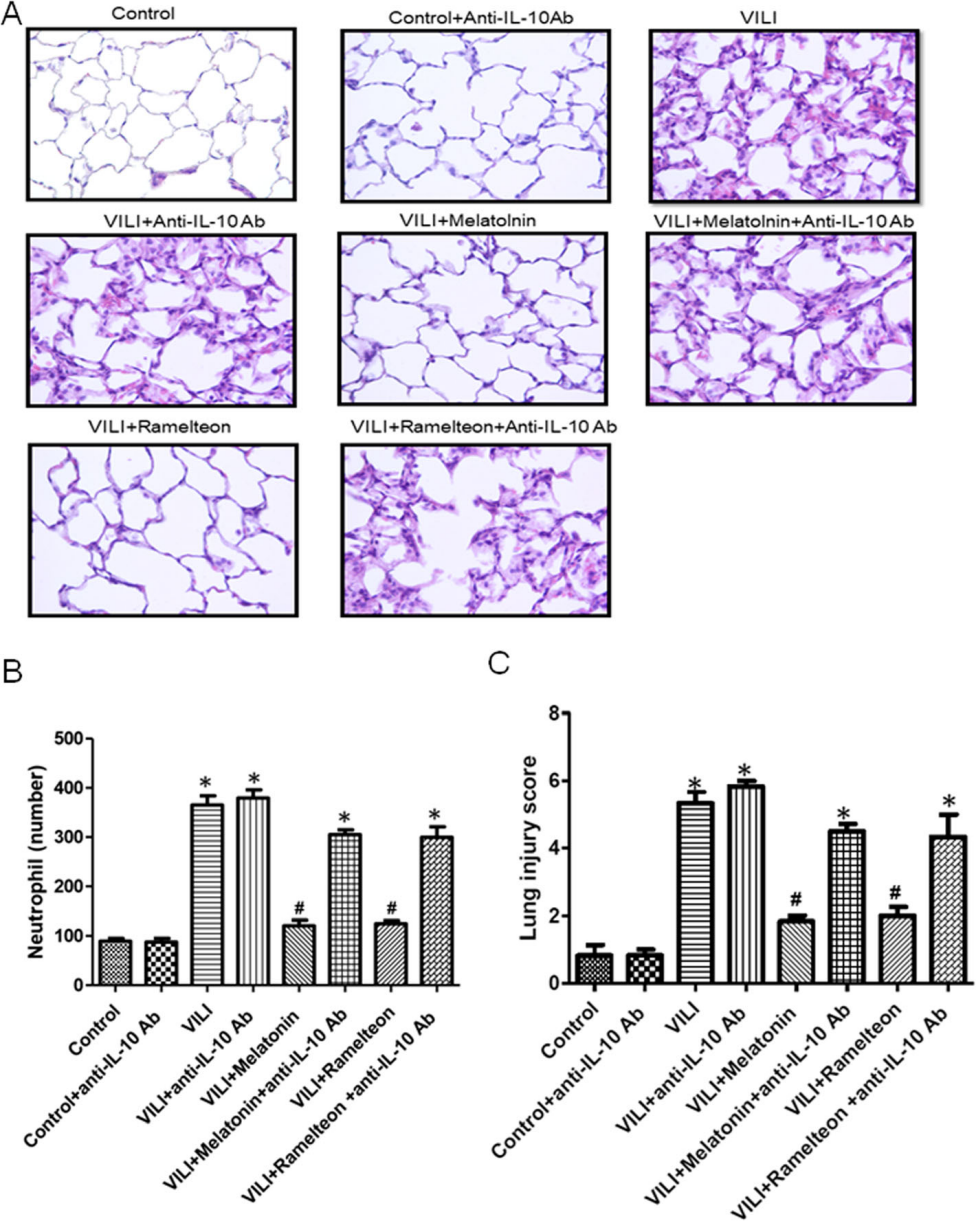
Effect of an anti-IL-10 antibody on the melatonin- and ramelteon-mediated lung pathology. **a** Hematoxylin and eosin staining analysis of lung pathological injury. **b** The numbers of neutrophils per high-power field, and **c** lung injury score. Representative photomicrographs were taken at a magnification of × 400. Histological evaluation of lung tissues showed that neutrophil infiltration and the lung injury score were increased in the VILI group. Melatonin or ramelteon treatment significantly attenuated these histopathological changes, but the protective effect of melatonin and ramelteon was abrogated by anti-IL-10 antibody treatment. The data are expressed as the mean ± SD (n = 6 per group). *Significantly different from the control (p < 0.05); *#*significantly different from the VILI group (p < 0.05)

As shown in Fig. 12, the control group exhibited normal lung tissue structures. In contrast, severe lung damage was observed in the VILI group, as indicated by extensive interstitial edema and leucocyte infiltration. Melatonin or ramelteon treatment significantly reduced the histological changes (Fig. 12a), neutrophil infiltration (Fig. 12b), and lung injury scores (Fig. 12c) in the VILI group. However, the protective effects were abolished by anti-IL-10 antibody treatment.

### Effect of an anti-IL-10 antibody on the melatonin- and ramelteon-mediated apoptosis and NF-κB signaling pathway

**Fig. 13:**
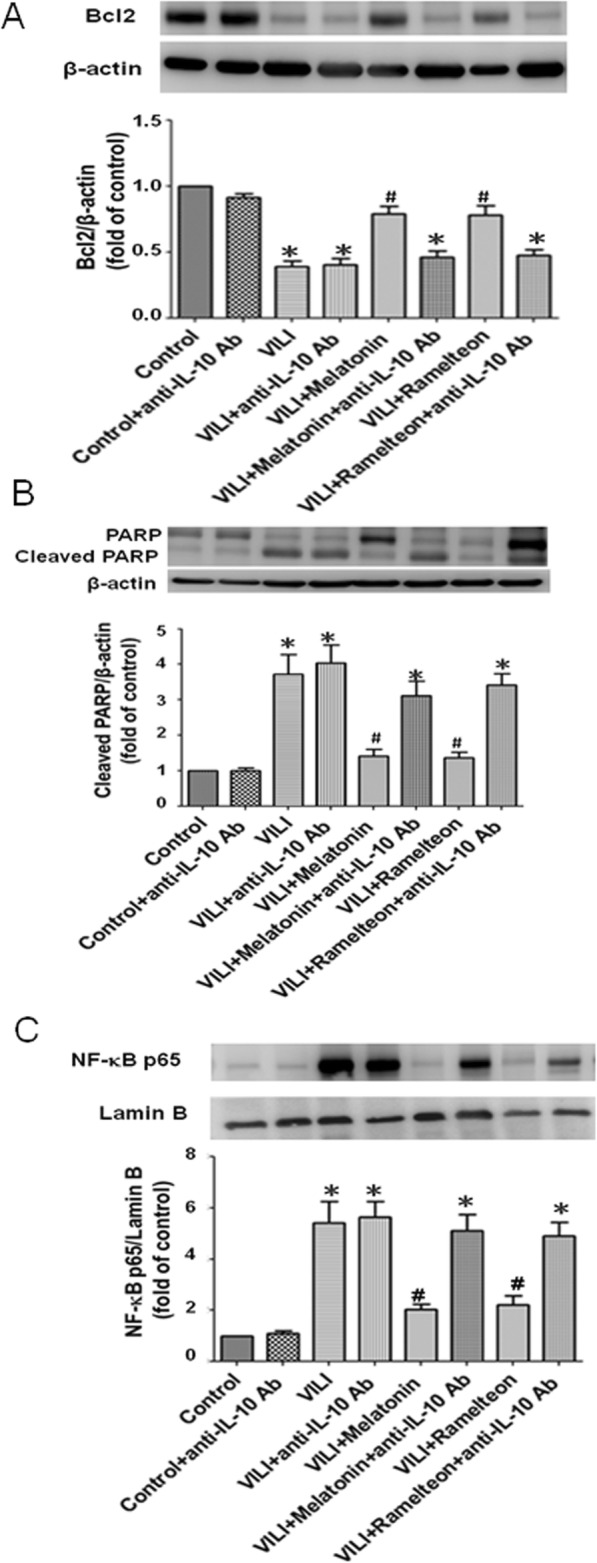
Effect of an anti-IL-10 antibody on the melatonin- and ramelteon-mediated apoptosis and NF-κB signaling pathway. **a** Bcl2, **b** cleaved PARP,and **c** NF-κB p65 levels in the lung tissue were determined by western blotting. Lamin B and β-actin served as loading controls for nuclear and cytoplasmic proteins, respectively. Melatonin or ramelteon treatment increased Bcl2, and reduced cleaved PARP and NF-κB p65 levels in VILI. When anti-IL-10 antibody was added, the protective effect was blocked. The data are expressed as the mean ± SD (n = 6 per group). *Significantly different from the control (p < 0.05); ^*#*^significantly different from the VILI group (p < 0.05)

The Bcl-2 protein levels (Fig. 13a) in the lung tissue were substantially lower in the VILI group than the control group. However, they were significantly increased upon melatonin or ramelteon treatment. The protective effect was abolished by treatment with anti-IL-10 antibody. The expression of cleaved PARP in the lung tissue was higher in the VILI group than the control group, but melatonin or ramelteon treatment attenuated the increased expression of cleaved PARP in the VILI group. Similarly, the protective effects of melatonin and ramelteon were abolished by treatment with anti-IL-10 antibody (Fig. 13b). The protein level of NF-κB p65 in the nucleolus was significantly increased in the VILI group compared with the control group. Melatonin or ramelteon treatment reduced nuclear NF-κB p65 protein expression. Treatment with anti-IL-10 antibody counteracted the protective effect of melatonin and ramelteon (Fig. 13c).

## Discussion

This study demonstrated that VILI significantly increased lung edema, neutrophil infiltration, inflammatory cytokine production, oxidative stress, apoptosis, IκB-α degradation, nuclear translocation of NF-κB, and tissue injury. These effects were significantly attenuated by treatment with melatonin or ramelteon. However, the protective effect of melatonin or ramelteon was blocked by the administration of luzindole or an anti-IL-10 antibody. These findings indicate that melatonin receptors mediate the protective effects against VILI and that the underlying protective mechanism of ramelteon occurs via the enhancement of IL-10 production.

Oxidative stress contributes significantly to the pathogenesis of ALI/ARDS [19]. Melatonin is a strong anioxidant that scavenges ROS, decreases MDA levels, and stimulates the synthesis of antioxidant enzymes. The protective effect of melatonin against oxidative stress may occur via the activation of melatonin receptors, which can stimulate the production of a variety of antioxidative enzymes through several signaling pathways [20, 21]. Nevertheless, recent studies also demonstrate that melatonin can act as a prooxidant under certain conditions [22]. Generally, the prooxidant effects of melatonin have been reported in vitro cell culture systems and dependent on concentration, cell type, and the duration of the treatment [22]. The antioxidant capacity of melatonin has been well established both in vitro and in vivo, while its prooxidant property has been observed at the same time [14, 22–25]. The relevance of this finding is unclear, therefore, more studies addressing this issue are necessary.

Our data demonstrate that melatonin and ramelteon treatment suppressed oxidative stress, as reflected by the attenuation of protein carbonylation in lung tissue and serum MDA levels in VILI. In addition, melatonin and ramelteon treatment attenuated the VILI-induced increase in neutrophil infiltration in the lung tissue, as evidenced by the reduced numbers of neutrophils and MPO-positive cells. This attenuation reduced the production of proinflammatory cytokines and free radicals by activated neutrophils. Therefore, the antioxidative and anti-inflammatory effects of melatonin and ramelteon appeared to attenuate lung edema, as shown by the reduced W/D and LW/BW ratios, as well as reduced protein concentration in BALF. However, the antioxidative and anti-inflammatory effects of melatonin and ramelteon were significantly decreased by luzindole treatment. These results demonstrate that melatonin receptors play an important role in the antioxidative and anti-inflammatory effects of melatonin and ramelteon. As previous reports, the antioxidative and anti-inflammatory effects of ramelteon were reversed by the melatonin receptor antagonist luzindole [12, 26], we suggest that ramelteon mediates its protective effect of VILI mostly through the melatonin receptor.

ROS have been shown to promote the apoptotic response in various physiological processes [27]. Epithelial cell apoptosis also occurs in ALI/ARDS, and can potentially be limited by blocking apoptosis pathways [19, 27]. Caspase-3 is a key player in the execution phase of apoptosis, and PARP is cleaved by caspase-3 during the process. Reducing the cleavage of PARP can prevent ALI development [27]. Bcl-2 can protect cells against apoptosis, and evidence suggests that its overexpression can inhibit apoptosis in response to a variety of stimuli. This inhibition of apoptosis involves the modulation of caspase-3 and PARP degradation [28].

Hsp70 is an important type of stress-induced protein and is also an antiapoptotic protein that protects tissues from cytotoxicity induced by oxidative stress and other stress conditions [29]. Previous studies have implied that ALI leads to the downregulation of Hsp70 expression in the lungs [18]. Another study demonstrated that enhanced Hsp70 expression attenuates apoptosis in ALI [30]. Moreover, HSP70 overexpression inhibits caspase-3 activation and PARP cleavage [31, 32].

Melatonin has been shown to inhibit apoptosis by decreasing caspase-3 and increasing Bcl-2 expression in injured lungs [14]. Additionally, melatonin enhances the expression of heat shock proteins, such as Hsp70, preventing renal damage and arrhythmogenic myocardial remodeling during unilateral ureteral obstruction [29]. The antiapoptotic effect of melatonin is mediated by melatonin receptors, and luzindole abolishes their activity [4]. A vast amount of evidence has demonstrated that the antiapoptotic effects of melatonin involve the membrane melatonin receptors. Sinha et al. showed that the activation of caspase-3 in hypoxic-ischemic injury occurs in parallel to the downregulation of melatonin receptor expression. The activation of caspase-3 is reduced by the administration of melatonin, while luzindole effectively blocks the melatonin-induced inhibition of caspase-3 activation [33, 34].

In addition, melatonin activates Bcl-2 in the mitochondria, which antagonizes bax and thereby inhibits apoptosis via interactions with melatonin receptors [35]. Furthermore, the activation of melatonin receptors increases the expression of Hsp70 [36]. As shown in the present study, the antiapoptotic and antioxidant effects of melatonin seem to be dependent on melatonin receptors. Similar to the effect of melatonin, the present results show that ramelteon has the ability to modulate ROS production and Bcl-2, caspase-3, PARP, and Hsp70 expression, which results in the attenuation of VILI.

Activation of the NF-κB signaling pathway is central to the pathophysiology of the inflammatory response, and NF-κB can be activated by oxidative stress, bacterial endotoxins, and cytokines. The functional importance of NF-κB in inflammation is based on its ability to regulate the promoters of multiple inflammatory genes, including TNF-α, IL-1β, IL-6, and iNOS [37]. The inappropriate activation of NF-κB is implicated in the pathogenesis of ALI/ARDS [19]. Moniruzzaman et al. demonstrated that melatonin regulates the activation of NF-κB through the induction of the expression of its receptor protein [38]. Moreover, the expression and activity of iNOS are significantly upregulated by high tidal ventilation and correlate with lung edema in VILI. iNOS-deficient mice are protected from pulmonary edema in response to mechanical ventilation with high tidal ventilation [39].

Jung et al. demonstrated that melatonin treatment attenuates the protein expression of iNOS in rats with dimethylnitrosamine-induced liver injury [40]. Hsu et al. demonstrated that melatonin treatment attenuates iNOS expression, which is mediated by melatonin receptors, as luzindole treatment blocks the melatonin-induced decrease in iNOS expression [41]. The present study results show that melatonin or ramelteon treatment inhibits the expression of NF-κB and iNOS. The effects of melatonin or ramelteon may be mediated by binding to melatonin receptors since luzindole blocks the decreases in expression.

Proinflammatory cytokines play a pivotal role in ALI/ARDS [42]. For example, the elevation of cytokines and chemokines such as TNF-*α*, IL-1β, IL-6, and IL-8 is a feature that is common in ALI/ARDS [42]. In a previous report, melatonin was found to inhibit LPS-stimulated TNF-α, IL-1β, IL-6, IL-8, and iNOS production through a mechanism involving the attenuation of NF-κB activation. The effects of melatonin could be at least partially mediated by binding to melatonin receptors because luzindole attenuates melatonin-induced decreases in the production of proinflammatory cytokines [43]. Consistent with previous reports, we found that melatonin or ramelteon treatment decreased the levels of proinflammatory cytokines in VILI via melatonin receptors.

STAT3 is considered an important proinflammatory regulator. The activation of STAT3 may be a common signaling mechanism that contributes to the pathogenesis of ALI. The suppression of STAT3 activity has been shown to attenuate LPS-induced ALI [44]. STAT target genes contribute to the production of cytokines, chemokines, adhesion molecules, and other inflammatory mediators. IL-6 is a well-described STAT3 activator [45]. In the current study, the activation and inhibition of STAT3 phosphorylation occurred in parallel with IL-6 production. Furthermore, we also found that melatonin or ramelteon treatment inhibited IL-6 production and consequently decreased STAT3 phosphorylation, which was also mediated via melatonin receptors.

IL-10 is an anti-inflammatory cytokine in the context of both acute and chronic inflammation [46]. IL-10 has multiple protective effects and is known to alleviate ALI by reducing the activity of MPO, attenuating the production of TNF-*α*, IL-1β, IL-6, and MIP-2, suppressing NF-κB activation, and decreasing iNOS expression [2, 47, 48]. In an in vivo experiment, Hoegl et al. showed that the inhalation of IL-10 reduced the concentrations of MIP-2 and IL-1β in BALF and plasma induced by high-pressure ventilation [2]. Additionally, IL-10 increased the survival of animals [2]. IL-10 also inhibited ROS production, which in turn inhibited IκB-α degradation, decreased NF-κB activity, and hence decreased the expression of inflammatory cytokines [49]. Furthermore, IL-10 has also been shown to suppress the expression of iNOS and reduce the production of nitric oxide in various types of cells [50].

Lee et al. showed that IL-10 protects cultured rat type II epithelial cells from injury induced by mechanical stretching by decreasing apoptosis [51]. IL-10 has antiapoptotic effects since it has been shown to inhibit TNF-α-induced caspase activity and restore the impaired bax/bcl-2 ratio in articular chondrocytes [52]. Moreover, the Hsp70-driven downregulation of proinflammatory cytokine production is dependent on IL-10 [53, 54]. Wu et al. showed that the IL-10 expression induced by melatonin can improve ALI induced by heatstroke [55].

Melatonin receptors are tightly associated with the signal transduction of melatonin [56, 57]. In this study, we found that the protective effects of melatonin exerted against VILI are mediated by melatonin receptors. Melatonin- or ramelteon-induced IL-10 expression in VILI is mediated by melatonin receptors since luzindole treatment abrogates the effect. When we administered an anti-IL-10 antibody, the protective effects of melatonin and ramelteon against VILI were abrogated, resulting in increasing lung edema, TNF-α, IL-1β and IL-6 levels in BALF, NF-κB expression, and apoptosis in lung tissue, which indicated that melatonin receptors upregulated IL-10 production and improved VILI. However, further study is warranted to clarify how melatonin receptor agonist can enhance IL-10 production.

## Conclusions

Collectively, our results show that the melatonin receptor agonist ramelteon significantly ameliorated VILI by mitigating lung edema, the production of inflammatory cytokines and ROS, NF-κB signaling, and apoptosis. Furthermore, the protective effects of ramelteon were abolished by the administration of an anti-IL-10 antibody. Our investigation provides new insights into the underlying mechanisms of melatonin receptor-mediated protection against VILI via the upregulation of IL-10 production. These results could lead to novel treatment targets for VILI.

## Supplementary information

**Additional file 1: Supplemental Figure 1.** Effect of vehicle on the VILI -mediated lung injury. VILI significantly increased the lung weight/body weight ratio (A), W/D weight ratio (B), and lung injure score (C). Vehicle groups did not induce or exacerbate lung injury. The data are expressed as the mean ± SD (*n* = 6 per group). *Significantly different from the control (*p* < 0.05); ^*#*^significantly different from the VILI group (*p* < 0.05).

## Data Availability

All data are provided in the manuscript.

## Abbreviations

ALI: Acute lung injury
ARDS: Acute respiratory distress syndrome
BALF: Bronchoalveolar lavage fluid
Bcl2: B-cell lymphoma-2
Hsp70: Heat shock protein 70
LW/BW: Lung weight/body weight
MDA: Malondialdehyde
MPO: Myeloperoxidase
NF-κB: Nuclear factor-κB
PARP: Poly (ADP-ribose) polymerase
ROS: Reactive oxygen species
TNF-α: Tumor necrosis factor-α
VILI: Ventilator-induced lung injury
W/D: Wet/dry
