# Supplementary figures and images for: Melatonin receptor agonist protects against acute lung injury induced by ventilator through up-regulation of IL-10 production
Source: Respir Res. 2020 Mar 6;21:65. doi: 10.1186/s12931-020-1325-2 (PMC7059294; doi:10.1186/s12931-020-1325-2)

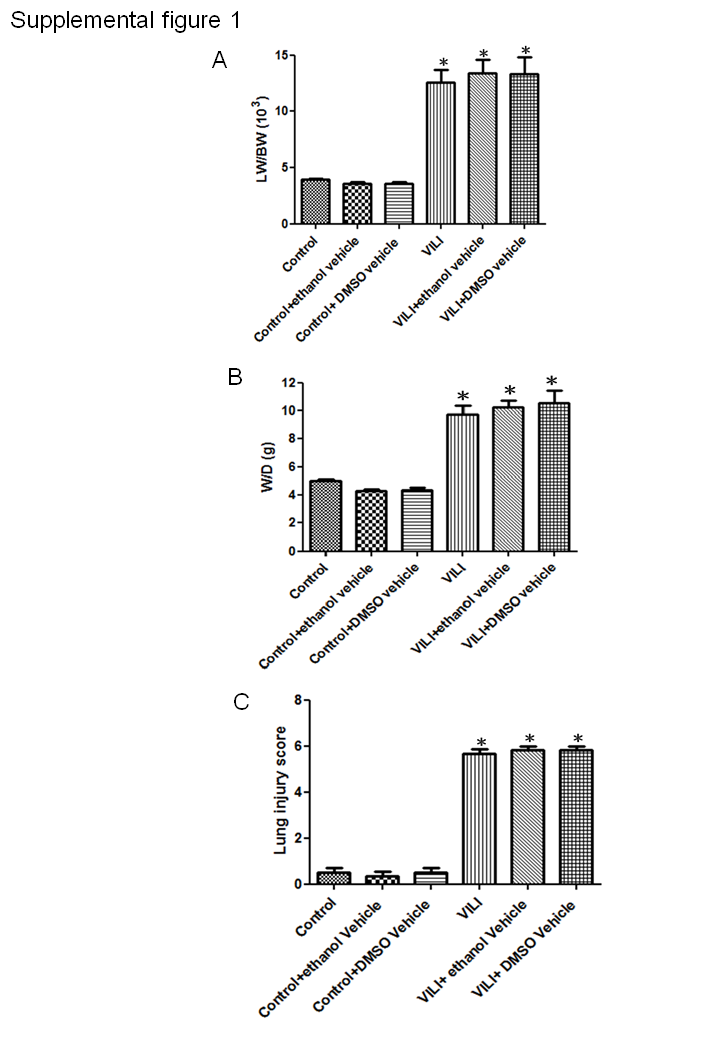

Supplement: Supplementary file 1 — Additional file 1: Supplemental Figure 1. Effect of vehicle on the VILI -mediated lung injury. VILI significantly increased the lung weight/body weight ratio (A), W/D weight ratio (B), and lung injure score (C). Vehicle groups did not induce or exacerbate lung injury. The data are expressed as the mean ± SD (n = 6 per group). *Significantly different from the control (p < 0.05); #significantly different from the VILI group (p < 0.05). [file 12931_2020_1325_MOESM1_ESM.tif]
